# The emergence of embedded structure: insights from Kafr Qasem Sign Language

**DOI:** 10.3389/fpsyg.2014.00525

**Published:** 2014-06-03

**Authors:** Itamar Kastner, Irit Meir, Wendy Sandler, Svetlana Dachkovsky

**Affiliations:** ^1^Department of Linguistics, New York UniversityNew York, NY, USA; ^2^Department of Hebrew Language and Department of Communication Disorders, University of HaifaHaifa, Israel; ^3^Department of English Language and Literature, University of HaifaHaifa, Israel

**Keywords:** sign language, prosody, embedding, syntax, nominalization

## Abstract

This paper introduces data from Kafr Qasem Sign Language (KQSL), an as-yet undescribed sign language, and identifies the earliest indications of embedding in this young language. Using semantic and prosodic criteria, we identify predicates that form a constituent with a noun, functionally modifying it. We analyze these structures as instances of embedded predicates, exhibiting what can be regarded as very early stages in the development of subordinate constructions, and argue that these structures may bear directly on questions about the development of embedding and subordination in language in general. Deutscher (2009) argues persuasively that nominalization of a verb is the first step—and the crucial step—toward syntactic embedding. It has also been suggested that prosodic marking may precede syntactic marking of embedding (Mithun, 2009). However, the relevant data from the stage at which embedding first emerges have not previously been available. KQSL might be the missing piece of the puzzle: a language in which a noun can be modified by an additional predicate, forming a proposition within a proposition, sustained entirely by prosodic means.

## Introduction

If a woman sits on a sofa and a man shows her a picture, can we say that *the man is showing the seated woman a picture*? We can in English and in many spoken languages; the participle *seated* allows us to express a secondary predicate which is subordinate to the main clause. As we started to investigate an as-yet undocumented young sign language from the town of Kafr Qasem in Israel we noticed an unexpected moderate tendency to use secondary predicates as noun modifiers. We regard this phenomenon as embedding, and situate it within two contexts. The first context is the overall emergence of structure in Kafr Qasem Sign Language (KQSL). The second is the question of how embedding and subordination may have evolved in natural language in general, over the ages. The latter is still a mystery for the most part, since we do not have documentation of early enough stages in the life of a language. The rise of embedding in KQSL, caught at a relatively early stage of development, could provide a clue to the initial stages of this phenomenon, and shed some light on the rise of syntactic complexity in general.

Since this is the first published work on KQSL, we begin by introducing the language, focusing on relevant historical and social aspects. We report on a brief study verifying that KQSL is not related to other sign languages in the region. We then describe our methods. The following sentence presents the results and analysis of the structures which we regard as embedded. The embedding findings arose while eliciting sentences for an investigation of word order, and we touch on this to put our main finding in perspective. To put this issue into a historical perspective, in Section Discussion: Embedding by Hand and by Mouth we compare possible origins of embedding that have been proposed for spoken languages with the KQSL findings. We summarize our findings and their theoretical relevance in the conclusion.

## Kafr qasem sign language

Sign languages arise spontaneously in communities of deaf people, and are not related to (though are possibly influenced by) the ambient spoken languages. In some countries, signers have by and large converged upon a single sign language, used by the deaf population throughout the country. Thus, deaf people in America use American Sign Language (ASL), while deaf people in Britain use British Sign Language (BSL), two mutually unintelligible languages. In Israel, the established language of the deaf community of about 10,000 signers is Israeli Sign Language (ISL). Yet Israel is home to a number of other sign languages which have arisen there over the past century, languages used by smaller, sometimes insular communities with an unusually high percentage of congenital deafness. Such languages are termed *village sign languages* (Meir et al., 2010; Nyst, 2012; Zeshan and de Vos, 2012) present other terms used in the literature to refer to these communities). Two village sign languages have already been documented in Israel. In the Bedouin village of Al-Sayyid, a community of about 4000 members of whom 130 are deaf, a sign language arose about 80 years ago (Al-Sayyid Bedouin Sign Language, ABSL), and, for more than a decade, has been the focus of intensive anthropological (Kisch, 2000, 2007, 2012) and linguistic investigation (e.g., Sandler et al., 2005; Aronoff et al., 2008; Meir et al., 2013; Sandler et al., in press). In the Jewish community of the sub-Saharan town of Ghardaia, Algeria, another sign language emerged (Algerian Jewish Sign Language, AJSL). When the community members left Algeria and immigrated to Israel and France, they brought the sign language with them and continue to use it to this day (Lanesman and Meir, 2012a,b). To the list of sign languages being investigated in Israel, we now add KQSL.

The town of Kafr Qasem lies in the so-called Triangle area of Arab towns in central Israel and has existed for 350 years. Of its 20,000 residents, approximately 100 are deaf. From reports and interviews with residents of the town we estimate that the language is four generations old. Our team has been in touch with the local deaf community since early 2010, gathering social and historical data and analyzing the language's phonology, lexicon and syntax. This work has led us to conclude that KQSL is an independent language, worthy of study both for its own sake and in comparison to other languages. We give the results of a lexico-statistical study supporting this view, after providing some historical context.

### History and sociolinguistic context

From what we have been able to uncover, an 80-year-old woman is the oldest living signer of KQSL. She is known to have had deaf aunts and uncles, placing our estimate for the age of the language at just under 100 years. Until the 1960s, the number of deaf people in the village was about 12. A rapid increase in the general population resulted in an increase in the number of deaf individuals, numbering about 30 by 1980, and over 100 today (Meyad Sarsur, pers. commun. 2013).

We do not possess detailed records of the deaf population over the last century. The history of the local community, gathered through interviews with people in the community, is as follows. A deaf woman from the south of the country married a hearing man from the village over 100 years ago, later giving birth to a number of deaf children. In the 1920s, 1930s and 1940s there were 1800 inhabitants in the village, out of whom 12 were deaf (7 male, 5 female). By the early 1980s the number reached 31 (14 male, 17 female). The rise in the number of deaf children led to the opening of a class for deaf children in the local school in 1979. Although the first teacher was a non-signer, in 1985 a teacher who used signing started working in school, introducing ISL vocabulary into the educational system there. Since 1993, cochlear implants have been available through the Israeli health system; around 30 children have been implanted. According to one of our deaf consultants, the parents of these children “reject the use of sign language.” A number of parents of deaf children founded a local association in 1995, paving the way for a deaf club for afternoon activities with sign language which opened in 1996. Today the town has a number of educational programs available for deaf children: a local branch of *MICHA*, the Israeli preschool system for young deaf children; Learning Centers; a kindergarten; elementary school classes in the nearby town of Jaljulia; and classes for deaf and hard-of-hearing children in the local junior high and high school.

From our interviews with deaf and hearing people who are 60 years and older, we have learned that the older deaf people in the village spent a lot of time with each other as a group in their childhood. They have a sense of “togetherness” that has persisted to this day. There are many social meetings held at the house of one of the older women which serves as the local gathering place for deaf people of all ages, situated on a street nicknamed “the deaf neighborhood.” Some of the deaf people are married, usually to a hearing spouse, though there is at least one deaf-deaf marriage in the younger generation. The hearing spouses, siblings, children and neighbors of deaf people communicate with them in sign. Hearing people that we interviewed report that this has been the practice for as long as they remember, which goes back about 60–70 years. Since contact with the Jewish deaf community and the general educational system for the deaf in Israel began around the late 1970s, we assume that the sign language that emerged in the village up to that point developed independently of ISL. We have found no evidence of contact with the better studied village sign language in Israel, ABSL, over the years. This is not surprising, due to geographical and cultural distances between the two. Even today, contact between people from the two communities is very rare. We therefore conclude that KQSL developed as an independent local sign language. This conclusion is corroborated by the lexical comparative study reported below.

However, deaf people in Kafr Qasem who entered the educational system in the 1980s and onwards have been heavily influenced by ISL, and many of them find it hard to understand the older people who use only KQSL. In order to document and describe the linguistic structure of KQSL, it is necessary to study the language of those signers who have had little contact with ISL over the years, and who use KQSL regularly as their main means of communication. The study reported here is based on the sign production of six such KQSL signers.

### Evidence for KQSL as an independent language

One clear indication that two languages are unrelated is the existence of two different lexicons. In fact, one of our first impressions of KQSL was that its vocabulary is unlike that of ISL or ABSL, and we therefore had to be aided by an interpreter to interact with signers there. In the KQSL lexicon, we have found signs for tangible objects, abstract concepts, actions and feelings. Many of these are directly related to the local culture. For instance, KQSL has two signs for the concept *sheep*, each referring to a different type of sheep, a distinction important for a community that used to engage in herding. The lexicon also includes signs that can be regarded as function words, such as negators, signs denoting quantity and signs denoting degree.

In order to determine the degree of overlap between KQSL and the neighboring sign languages, we follow previous work in comparing the lexicons of sign languages and their dialects (McKee and Kennedy, 2000; Guerra Currie et al., 2002; Hendriks, 2008; Al-Fityani and Padden, 2010). The methodology is based on comparing the relative resemblance between signs in different languages that denote the same concept. To this end we require definitions of what *identical* signs and what *similar* signs are, and so a word on sign language phonology is in order.

**Figure 1:**
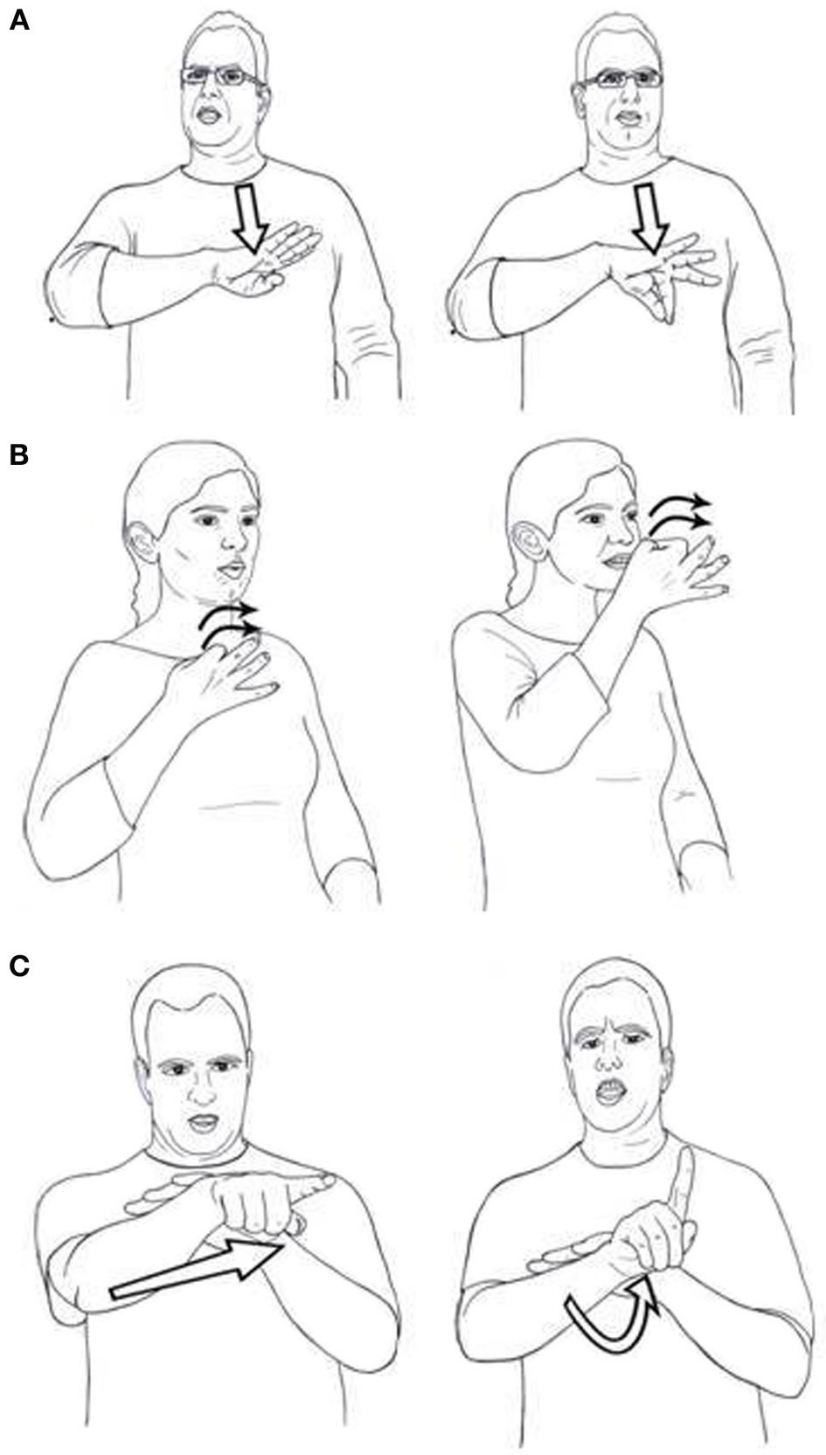
**(A)** Two signs distinguished by handshape: deprive and profit. **(B)** Two signs distinguished by location: well-being and curiosity. **(C)** Two signs distinguished by movement: escape and betray.

Contrastive features in established sign languages are hierarchically organized into a number of major phonological categories: handshape, location and movement (see Sandler, 2012 for an overview). Although there is some debate regarding the status of palm orientation in the system, i.e., whether it is a fourth major category or subordinate to handshape (Sandler and Lillo-Martin, 2006, p. 156), this theoretical point is not of great import here; features of orientation are clearly distinctive in sign languages, and this suffices for our purposes. That these categories contain contrastive features can be readily seen through examination of minimal pairs, using ISL as an example. In ISL, the signs deprived and profit (Figure 1A) are distinguished by features of the two handshapes 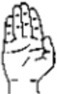 and 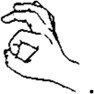 This is a minimal pair, because the locations and movements are the same in the two signs, which are distinguished by handshape alone. The ISL signs well-being and curiosity (Figure 1B) are minimally distinguished by features of location (chest vs. nose respectively), while escape and betray are distinguished by movement alone, straight for escape, and arc for betray (Figure 1C).

**Figure 2:**
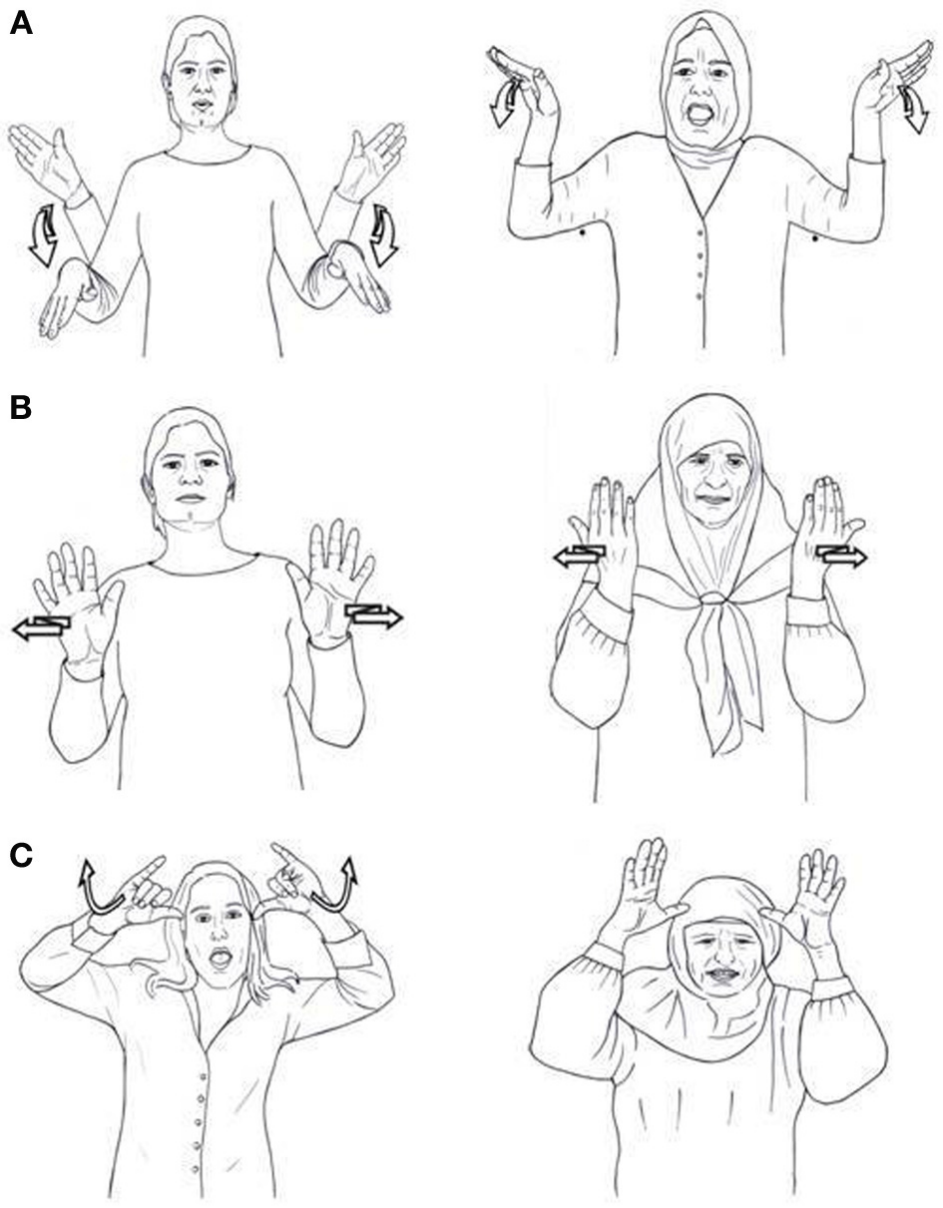
**(A)** Two identical signs in ISL and KQSL: bird. **(B)** Two similar signs in ISL and KQSL: television. **(C)** Two different signs in ISL and KQSL: cow.

These phonological features are used when comparing the lexicons of different sign languages in order to determine the degree of overlap between them. Following McKee and Kennedy (2000, p. 51), we define *identical* as signs which are pronounced with the same handshape, location, movement and orientation. *Similar* signs differ in only one of these parameters. Comparing ISL with KQSL signs, we find a few signs in the two languages that are identical; they share all four components, as is illustrated by the sign for “bird” in Figure 2A. The signs for “television” (Figure 2B) are similar; they have the same handshape, location and movement, but differ in orientation. The signs for “cow” (Figure 2C) are different; they differ on more than one phonological component. They have a different handshape and a different movement.

Even sign languages that we know to be genetically unrelated might display substantial similarities in vocabulary. Previous work has shown that unrelated sign languages normally display between 20 and 30% overlap in their lexicons (McKee and Kennedy, 2000). For example, ASL shares 26.3 and 24.5% identical signs with two languages which are genetically related to each other but not to ASL itself, namely BSL and Australian Sign Language, respectively. Including similar signs in the tally raises the rates to 32.6 and 32.7% (McKee and Kennedy, 2000, p. 53).

Guerra Currie et al. (2002, p. 228) obtained similarity ratings of 38% in 112 sign pairs when comparing the distantly related Mexican Sign Language and French Sign Language; 33% similarity in 89 pairs between the culturally linked, but not genetically related, Mexican Sign Language and Spanish Sign Language; and 23% similarity in 166 pairs between the unrelated Mexican Sign Language and Japanese Sign Language.

**Figure 3:**
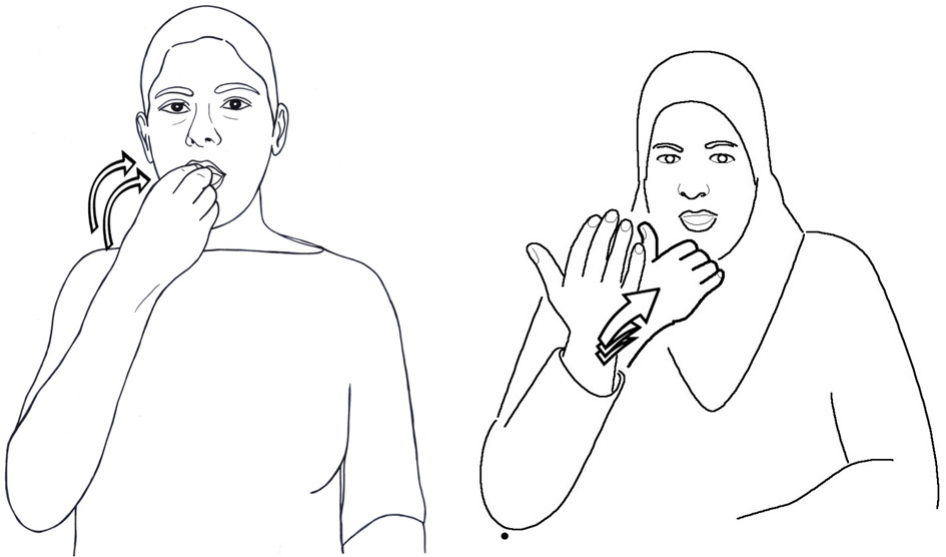
**eat in ISL and ABSL**.

This state of affairs, in which unrelated sign languages show such similarities, is attributed to iconicity, pervasive in the sign language lexicon. Two signs denoting the same concept in two different languages may represent iconically the same aspect of the concept being described. In such cases, they will display similarity in form, whether or not the two languages are related. For example, a sign for “eat” might represent food going into the mouth. In this sign, the location of the sign will be the mouth and the hand will move toward the mouth. The shape of the hand might vary from language to language, as it does in ISL and ABSL for example, shown in Figure 3. According to McKee and Kennedy's criteria, these two signs are *similar*. However, this similarity does not necessarily reflect a genetic relationship between the two languages. Even when the features of all four parameters are identical in two sign languages, as they are for eat in ISL and in ASL, one would not wish to use this coincidence as evidence for proximity on a sign language family tree. The two signs may be identical because they are built on the same mental image representing the concept. It is for this reason that a percentage as high as 30% similarity between the lexicons of two sign languages should not be interpreted as reflecting a family relationship. In contrast, British, New Zealand, and Australian Sign Languages are very closely related, with 82% identical signs from a Swadesh list, and 98% similar signs (McKee and Kennedy, 2000; Johnston, 2003).

**Figure 4:**
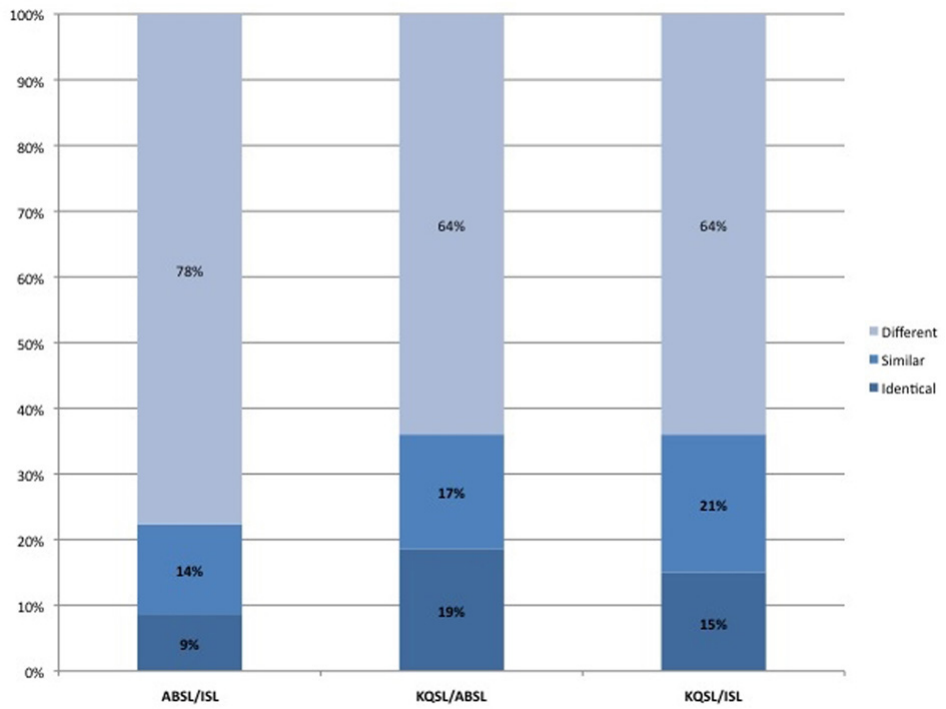
**Comparison of lexical similarity between ISL, KQSL, and ABSL**.

Returning to our comparison of KQSL, ISL, and ABSL, we have been using an adapted Swadesh list of concepts for comparison of different dialects and signers (we added a number of concepts that are likely to exist in all sign languages of the region, such as “Jerusalem”; the list is given in Supplementary Materials). Using this list we conducted three pairwise comparisons of elicited citation forms between ISL, KQSL, and ABSL. Coding was done by the first author and checked by the second author and two deaf consultants. Cases of disagreement were discussed until a consensus was arrived at. We compared 161 pairs of signs that exist as lexical signs in both KQSL and ABSL, finding a 19% overlap in identical signs which rose to 36% when similar signs were included. The overlap is similar when comparing KQSL and ISL: of the 186 pairs of signs that exist in both languages, 15% show overlap when considering identical signs and 36% overlap when including similar signs. The comparison of 161 pairs of signs in ABSL and ISL showed lesser degree of overlap: about 9% overlap with identical signs and 23% overlap when similar signs are included. The results of this comparison are given in Figure 4.

**Figure 5:**
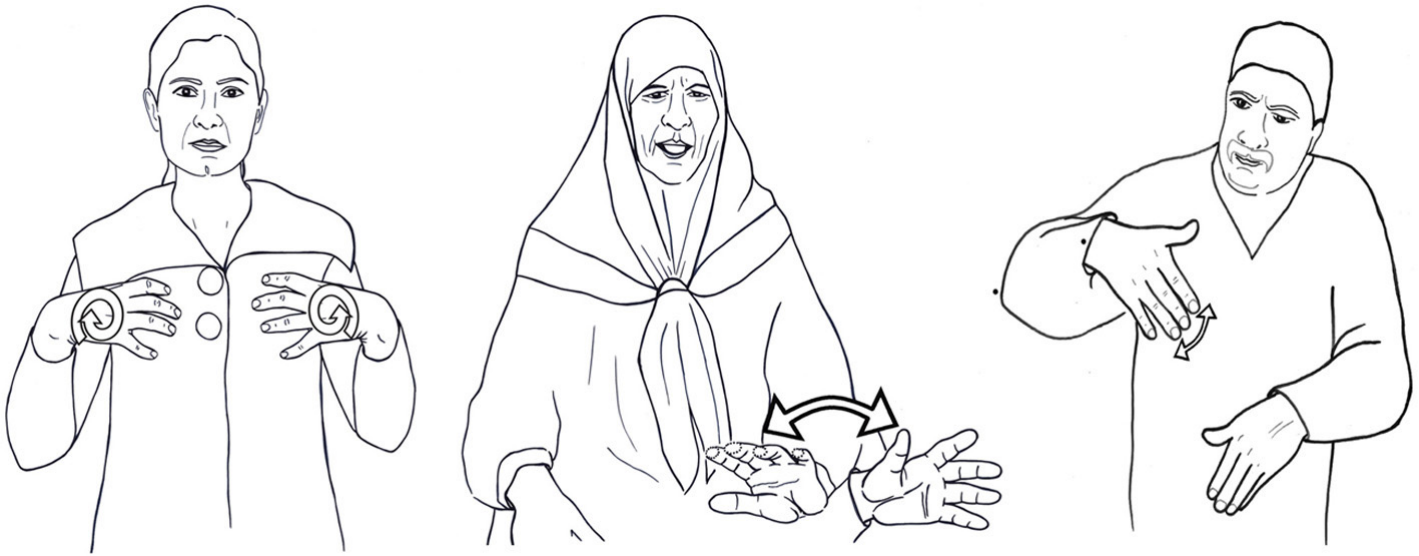
**The sign for sheep in ISL, KQSL, and ABSL**.

This suggests that, from a lexico-statistical point of view, KQSL and ISL are no more related than ASL and BSL are to one another (31% identical in the latter case). In spite of cultural similarities between the communities of KQSL and ABSL, their lexicons show a very similar degree of overlap. In some cases, similarity or identity between pairs of signs in these two languages may be partly due to some shared aspects of their culture, which are represented iconically by their vocabulary. For example, signs for “man” in KQSL and ABSL take the moustache to be the distinguishing feature of a man. This is a typical characteristic of men in Arab communities in the region, and is reflected in the choice of the mental image underlying the signs in both languages. In ISL, as in many European based sign languages including ASL, the sign for “man” is articulated on the forehead, maybe iconically representing a cap. The sign for “sheep” in ISL represents a wooly body, while the KQSL and ABSL signs represent the wobbly tail of the sheep, a very noticeable feature if you are a shepherd walking behind your herd (see Figure 5). Though the two signs are different, they might be regarded as similar since they differ only in orientation: in ABSL the fingertips are oriented downwards whereas in KQSL the fingertips point forward. Yet their similar form may reflect shared cultural practices rather than linguistic affiliation.

In spite of shared cultural practices and iconicity, the lexicons of KQSL and ABSL are different in about 65% of the items in our list, as are the lexicons of KQSL and ISL. From interviews with people in the Kafr Qasem and Al-Sayyid communities we learned that contact between members of the communities has been very rare and sporadic, and contact between deaf ISL signers and the older members of the KQSL deaf community has also been limited. Since the lexicons are different and the historical and social backgrounds are different, it seems reasonable to conclude that these are different languages with potentially different grammatical characteristics. Next we turn to the details of our study.

## Methods

### Participants

Six native KQSL signers participated in the study, five deaf and one hearing. The participants were of the 2nd and 3rd generation of Kafr Qasem deaf, with ages ranging between 42 and 67 (*M* = 52.7), four female and two male. Our group included one father-daughter pair. The results come from two of the female participants and one of the male participants. All participants received an explanation in sign language (ISL, which was then translated to KQSL by a member of the community) about the goals of the research and the methodological procedure. Only those participants who gave their consent to participate were included in the study.

### Materials

We used 30 short clips originally compiled for Sandler et al. (2005). The elicitation material included 13 intransitive sentences, 13 monotransitive sentences and four ditransitive sentences (the list is given in Supplementary Materials). Of the monotransitive sentences, eight portrayed a person acting on an object (e.g., a man washing a plate) and five portrayed an interaction between two humans (e.g., a girl combing a woman's hair). All four ditransitive sentences had two animate participants (e.g., a woman handing a shirt to a man).

### Procedure

Participants viewed these video clips on a laptop, one clip at a time, and then described them in sign language to another native signer seated across from them. The interlocutor was asked to choose one of three pictures portraying the scene on a printed page, in order to verify comprehension. For example, if the video showed a woman giving a shirt to a man, the page included a picture of a woman giving a man a shirt, a picture of a man giving a woman a shirt and a picture of a woman looking at a man. If an incorrect picture was chosen, the signer was asked to produce the sentence again. Some of the subjects signed the same sentence more than once of their own accord. Conditions were pseudo-randomized in advance, creating two lists with different item orders, so that each participant was shown the stimuli in one of two orders.

### Prosodic analysis

The elicited material underwent a preliminary gloss by a deaf research assistant, a native signer of ISL who has been in contact with KQSL signers for a number of years. Next, a quadrilingual consultant (KQSL, ISL, Arabic, and Hebrew) watched all elicited utterances and was recorded translating each one into Hebrew. This consultant, a trained ISL interpreter, is fluent in Arabic, KQSL, ISL, and Hebrew but was more comfortable volunteering simultaneous translations into Hebrew than sign-by-sign glosses. The preliminary glosses were compared to the translations provided, at which point the authors then discussed the best way to gloss each sign. Once agreement was reached, the gloss for each utterance was checked with the consultant once more to reach the final version reported here. Data were glossed and coded using the ELAN annotation system, which also provides the time windows for each annotation, allowing us to measure sign duration^1^.

The elicitation and glossing procedures were designed to analyze basic clause structure in KQSL, particularly word order. However, the first step toward defining clause structure is to define clause boundaries. In a language that has not been previously studied, this is not a trivial matter, since there is no pre-existing information regarding properties of clauses in the language. A dilemma regarding parsing of a stretch of discourse into clauses immediately arises when two signs denoting actions occur in the same response, as in: man sit get-up. How should this stretch of signs be analyzed? As a coordination of two clauses, e.g., “the man sat down and then stood up,” or maybe as one clause containing a main predicate and a secondary predicate, as in “the seated man (the man who was sitting) stood up”? Since we do not know anything about clause structure in the language, the initial analysis cannot be based on syntactic properties. In such cases we found that prosodic cues are very helpful.

In previous analyses of clause structure in unstudied sign languages (ABSL, Sandler et al., 2005; Padden et al., 2010) and ISL (Meir, 2010), a method was developed for determining clause boundaries based on the semantics and the prosody of the signs. Semantically, a clause is a unit containing a predicate, and associated signs are determined by thematic roles associated with the predicate. Prosodic cues, such as shifts in the rhythm marked by a pause or lowering of the hands, together with a change in head or body position, determine the boundaries of an intonational phrase, which often corresponds to a clause (Nespor and Vogel, 1986). Furthermore, prosodic cues also mark constituents within a clause, which often correspond to smaller syntactic constituents such as phrases.

The prosodic analysis employed here relies on the model of sign language prosody developed in Sandler and colleagues' work mainly on ISL (Nespor and Sandler, 1999; Dachkovsky and Sandler, 2009; Sandler, 2011; Dachkovsky et al., 2013). The prosodic cues in the list below were used as indicators of constituent boundaries, in accord with criteria developed in earlier work. These cues include manual timing and certain non-manual markers. These cues are typically combined at major prosodic constituent boundaries, i.e., intonational phrases. Specifically, increasing the salience of the final sign in a constituent through lengthening, holding the hands in place, or repeating the sign is accompanied by and aligned with a concomitant shift in head position and change of facial expression (Dachkovsky and Sandler, 2009; Dachkovsky et al., 2013). Smaller constituent boundaries (e.g., phonological or intermediate phrase boundaries) may be similarly marked by changes in hand rhythm and facial expression, but typically not by change in head or body position. Duration is increased at final prosodic constituent boundaries (Nespor and Sandler, 1999). An exception is constituent-final pronouns, which can cliticize onto their preceding hosts, and be observably reduced in duration (Sandler, 1999). Similar criteria have been used for determining constituent boundaries in previous work on ABSL (Sandler et al., 2005, 2011; Padden et al., 2010). The earlier literature does not provide measures of the duration of signs other than the final signs within constituents, a measure which we have found to be useful for the phenomenon under discussion in KQSL. Of the cues reported in the literature, the following are relevant for the present study:

1. Pause or hold. A pause in signing is defined as a period of transition during which the hands are relaxed. A hold is when the hands are held still at the final location for the sign (Liddell and Johnson, 1989). One or the other of these cues is typically found at the end of a prosodic phrase (Nespor and Sandler, 1999).
2. Change in body position. Among other uses of torso movement, such as role shift in a discourse, movement of the upper body can indicate the start of a new constituent (Boyes Braem, 1999 for Swiss German Sign Language, Fenlon et al., 2007 for Swedish Sign Language and BSL, Nicodemus, 2009 for ASL, Herrmann, 2009 for German Sign Language, Jantunen, 2007 for Finnish Sign Language, Van der Kooij et al., 2006 for Sign Language of the Netherlands, and see Ormel and Crasborn, 2012 for an overview).
3. Change in head position. The head turns to one side or the other, or moves forward or back^2^.
4. Lengthened sign duration and/or increased size beyond those of citation form.
5. Spread of facial expressions. While grammatical facial expressions, such as raised brows on yes/no questions, function as intonation (Nespor and Sandler, 1999) and typically characterize a whole intonational phrase (Liddell, 1980), some lexical signs are accompanied by a specific facial expression of their own. If this expression is spread to adjacent signs, we take this to be an indication that they belong to the same prosodic constituent (see discussion in Crasborn et al., 2008)^3^.

## Results

Here we report the word order results briefly, and go on to provide a detailed analysis of the embedded structure we found in our data.

### Word order

In total, 213 elicited utterances were recorded. Subject-Object-Verb (SOV) is the predominant order, occurring in over 63% of the responses, as in (1). OSV and SVO follow up with 19 and 14%, as in (2) and (3) respectively. We may conclude that SOV is the prevalent word order in KQSL. The remaining 4% are divided between less frequent orders such as SV, OV, and OVS (4); for a more detailed analysis of word order in KQSL compared to other languages see Meir et al. (in preparation)^4^.

(1) SOV: woman mother man see (1.2.04b)

    ‘A woman looks at a man’

(2) OSV: woman man book look (1.3.14)

    ‘A man shows a picture to a woman’

(3) SVO: father father wash-dishes plate (1.5.21)

    ‘A man washes dishes’

(4) Other: girl little push (1.2.03)

    ‘A girl drags a shopping cart’

### Embedding

#### Identifying an embedded predicate

Out of the 213 responses, 10 presented us with a challenge: they contained two predicates (two signs denoting an action or a property), yet the two signs seemed to differ in both their function in the string and in their prosody. We begin with an in-depth analysis of a representative utterance. Consider the following stretch of signs, describing a clip in which a man and a woman are sitting on the sofa, and the woman is looking at the man:^5^

(5) woman man sit eye^∧^look-at++.^6^(1.1.04)

The considerations we describe here were the same for each of the 10 examples analyzed. The first dilemma was whether the sign sit is predicated of both the woman and the man (“the woman and the man are sitting”), or whether it is predicated only of the man. Prosodic cues provided the answer.

After the sign woman, whose movement lasted about 400 ms, there was a hold in the movement of the hands (that is, the hands were held still at the final location of the sign) for about 250 ms. The end of the sign and the duration of the hold were aligned with a shift in both torso and head positions, the two bobbing slightly down and back up. This combination of features is typical of a prosodic boundary.

In contrast to this, there was no major postural change between man and sit. More importantly, there was no hold of the hands or pause after the sign man; rather, the signer immediately transitioned to the beginning of the following sign, sit. Instead, both the body posture and the behavior of the non-dominant hand linked man and sit within a single prosodic constituent. First, a change in head and body posture after woman characterized the two signs man sit. The sign sit is often accompanied by an upward movement of head and torso. In example (5), this upward posture of the torso starts on man, spreading regressively from sit to man. Additionally, the non-dominant hand, which is not used in the sign man but comes into use in sit, starts moving upwards toward the initial location of sit while the dominant hand still signs man. This process of Non-dominant Hand Spread within (but, crucially, not beyond) phonological phrases has been described for ISL and compared with external sandhi phenomena that take place within prosodic constituents (Nespor and Vogel, 1986; Nespor and Sandler, 1999).

The overall effect is one of a smooth transition between man and sit, as opposed to a marked break between woman and man. In other words, the prosodic pause between the sign woman and the sign man, and the assimilatory hand and body movement between man and sit, indicate that man sit is a constituent, to the exclusion of woman (in Figure 7 below, the relevant head and body postures are indicated with dotted lines). It is thus unlikely that sit modifies both woman and man (“a woman and a man are sitting”), but very likely that it modifies man. In still illustrations, the effects we describe appear quite subtle, but the movement of the body in actual signing makes both spreading and changes of postures more salient.

Next we examined the prosody of the transition from sit to the following sign, eye^∧^look-at. Here we see a break between the two signs, characterized by a change in head posture and body posture (head and body tilt to the left), dropping of the non-dominant hand, which was active in the sign sit, before the sign eye^∧^look-at, and a change in rhythm: eye^∧^look-at is slower. However, there is no pause or hold between the two signs, making the boundary less prominent than an intonational phrase boundary which typically delineates clauses (Nespor and Sandler, 1999), and we therefore do not interpret it as corresponding to a clause boundary.

What then is the structure of this utterance? The first two prosodic constituents are the two arguments of the transitive event: woman and man. The last constituent is the predicate (eye^∧^look-at). The order of constituents corresponds to the prevalent order in the language: it is SOV. However, we were baffled about how to analyze the verb sit that formed a prosodic unit with man, and did not itself mark the end of the clause. We then noticed that sit was much shorter in duration than the sentence-final verb, look-at, and much smaller in size. Let us make these measures explicit.

We use the term *shorter* signs to refer to the total duration (in milliseconds) of the sign production. We define the beginning of a sign as the point when the hands are fully in the handshape and orientation of that sign. The sign ends when they are no longer in that configuration. In the example above, sit lasted 170 ms whereas look-at lasted 400 ms. In a subsequent utterance (6), the same sign, sit, lasted 490 ms, showing that it is not the sign itself which is short but rather the specific production in sentence (5) above.

(6) woman sit. one^∧^girl small brush++. (1.1.22)

‘A woman is sitting. A girl is brushing her hair.’

**Figure 6:**
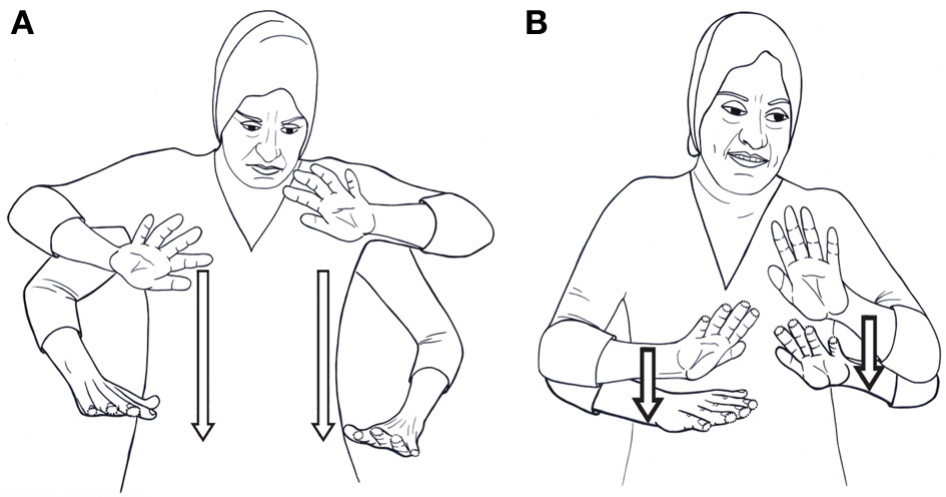
**(A)** sit (KQSL), non-small. **(B)** sit (KQSL), small.

When we say *smaller* signs, we refer to the amount of physical space “taken up” by the sign. This measure is more difficult to quantify, yet when a sign occurs in two responses, the size of the sign in the two productions can be compared. We illustrate this with the verb sit. In response (6), the two hands begin at chest level and are lowered to the hips, with each hand starting off with the palm faced outwards and the pinky finger near the ipsilateral shoulder (see Figure 6A). In example (5) above, however, the situation was slightly different. The dominant hand started off at chest level, while the non-dominant hand was a bit below it, such that the tips of the fingers in the non-dominant hand were in line with the palm of the dominant hand. The hands then moved slightly downwards, stopping just under the chest (see Figure 6B).

**Figure 7:**
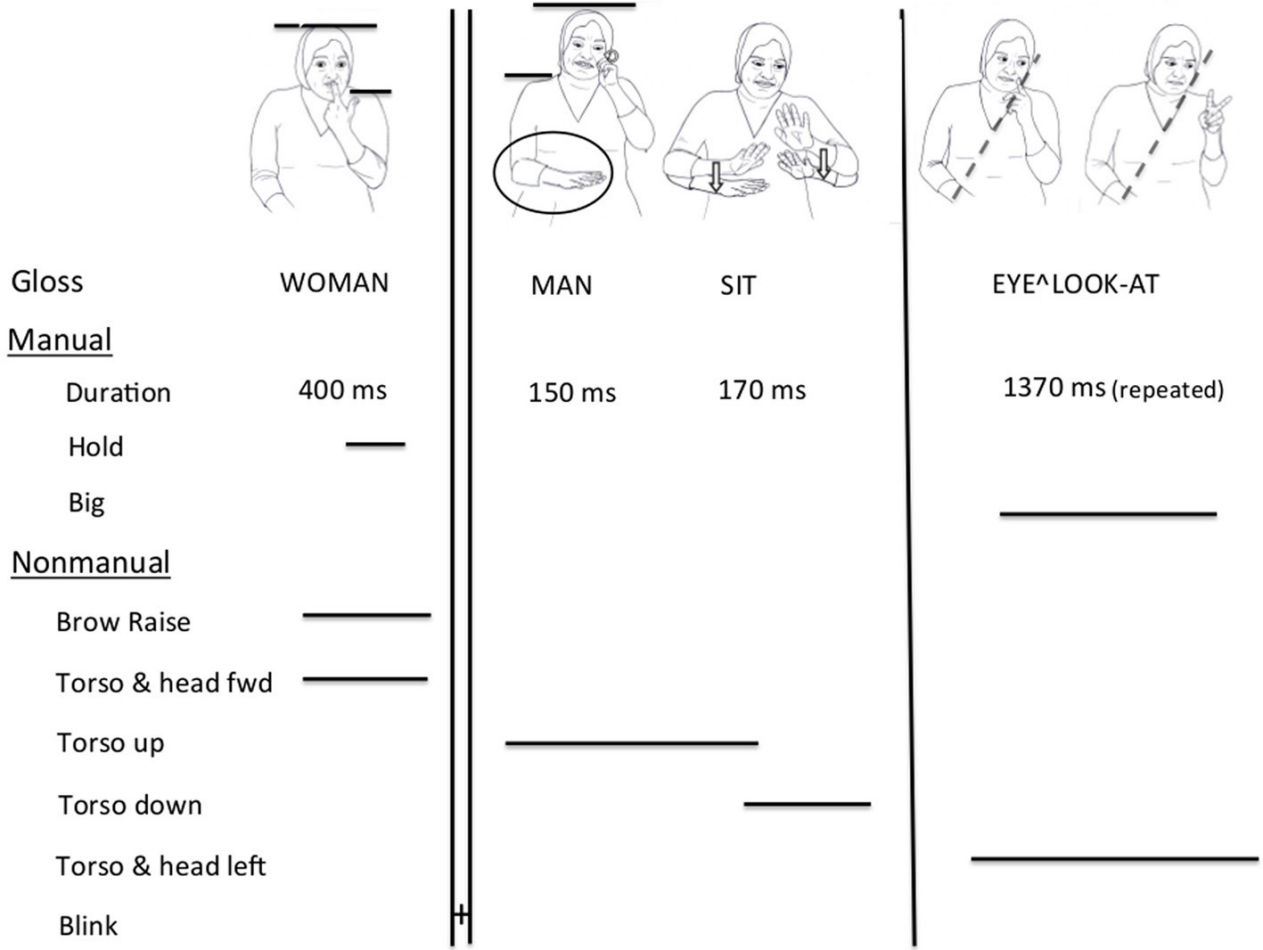
**Lexical and prosodic analysis of (5)**.

These two measures—shortened sign duration and reduced sign size—combine to give us a notion of phonological reduction. Taken together, the phonological reduction and the prosodic cues on and between woman, man, sit, and look-at indicate that in this production, sit does not behave as a main predicate in a clause: it forms one constituent with the preceding sign man, to the exclusion of the sign woman; there is a break between sit and look-at, signaled by change in head and body posture and change in rhythm, but it is not a typical major boundary, as there is no hold or pause between the two signs; and sit is much shorter than the other verbal sign in the clause, look-at. The full prosodic analysis of this clause is presented in Figure 7 below^7^. “Duration” notes the total length of each sign in milliseconds, “Hold” indicates that the hands were held in position at the end of a sign, and “Big” means that the size of the sign takes up more physical space than the citation form. As pointed out above, we take the prosody to signal syntactic constituency as well, and we therefore analyze sit as a secondary predicate in the clause, a modifier of man. An acceptable translation—confirmed by our consultant—would thus be “The woman is looking at the sitting (seated) man.” We conclude, then, that the response in (5) is a clause containing two predicates, a main predicate and a secondary predicate. The two predicates differ in their position in the clause and in their form: the main predicate occurs in clause final position, and is longer and larger in form. The secondary predicate follows a nominal sign and forms a constituent with it and is reduced in both size and duration. The structure of the clause is as follows:

(7) [woman] [[man sit] [eye^∧^look-at]]

‘The woman is looking at the sitting (seated) man’

#### Alternative hypotheses

Possible objections to the proposed analysis concern the difference in size and duration between the two predicates. It might be argued that look-at is longer than sit because it is in utterance final position, because it is verbal rather than nominal or because it is reduplicated. Let us consider these objections one by one.

Signs occurring utterance-finally tend to be longer and bigger (Nespor and Sandler, 1999). Accordingly, sit might be shorter not because it is embedded but rather because it is not in utterance final position. But since the prevalent word order in KQSL is SOV, there should be a strong tendency for the main predicate to occur in the clause final, prosodically prominent, position. How then can we show that the short duration of what we take to be an embedded predicate is indeed a signal of embedding rather than the result of its position in the utterance? Luckily, we found at least one example where the embedded predicate is in utterance final position while the main predicate is not:

(8) mother. ix mother woman sit. girl brush-other_450_++ brush_300_++, [mother sit_210_] (1.5.22)

‘A mother is sitting. A girl is brushing the sitting mother's hair.’

brush-other and brush are two variants of the “brushing” sign, with different hand orientations and locations; the former portrays a brushing action on an imaginary brushee and the latter on the signer herself. All sign durations are given for the non-reduplicated version, meaning one movement of the hands, even if the sign was reduplicated. For example, the first movement of brush-other in (8) took 450 ms, but in that utterance this movement was then repeated once more for a total duration of 800 ms for the entire, reduplicated sign. For the purpose of comparing main predicates and secondary predicates we calculated the duration of the main predicate without reduplication. In (8), the main verb, brush-other, is twice as long as the verb sit (450 and 210 ms., respectively), though sit is in final position and brush-other occurs in clause medial position. We conclude, then, that the relative duration and size of the predicate are indicative of its status as embedded or not.

The second objection concerns the status of grammatical categories in the language. It has been noted for various sign languages that nouns are reduced in size when compared to related verbs (e.g., Johnston, 2001 on Australian Sign Language; Hunger, 2006 on Austrian Sign Language; Kimmelman, 2009 on Russian Sign Language). It might be argued that sit is shorter than look-at as it is a nominal in a modifier position and not a “real” verb. We do not know enough about the differences between nouns and verbs in KQSL to address this question. Yet examples (19) and (20) below suggest that we should be cautious in drawing such a conclusion. The sign house appears in both examples, yet in (20) it is three times longer than in (20). This difference shows that the part-of-speech status of a sign cannot be the only factor determining its duration; prosodic factors play a role too. In the absence of a detailed analysis of the properties of nouns and verbs in the language, we prefer to couch our analysis in prosodic terms, since such an analysis is not based on a syntactic analysis which we do not have. The relationship between prosodic factors and parts of speech deserves future attention as we learn more about the behavior of nouns and verbs in KQSL.

Third, look-at is reduplicated whereas sit is not. Reduplication indicates aspectual marking in many sign languages (see Pfau et al., 2012 for a survey). The difference between look-at and sit might be argued to be that of aspectual marking, that is, morphological rather than prosodic. However, in four of the 10 examples we provide below, the main predicate is not reduplicated, yet the difference in duration between the two predicates is noticeable.

We therefore attribute the difference in size and duration between the two predicates first and foremost to the difference in their prosodic positions, which are related to their constituency affiliation. Whether other factors, such as parts of speech and aspectual modulation, also play a role in differentiating between the two predicates, is an issue that we leave for future research.

#### Other instances of embedding

As noted above, after analyzing the response in (5), we noticed in our data that such reduced predicates occur in nine additional responses, and are used by three out of the six signers. In eight of these nine cases, as in the example analyzed above, the reduced predicate forms one prosodic constituent with a preceding noun, which denotes one of the human participants in the clips (a man, a woman or a girl); that is, there is no break in any of the prosodic signals between the predicate and the preceding sign, motivating our analysis that these signs belong to one prosodic unit^8^. In several cases, the facial expression and/or body posture that accompany the second sign, the modifier, spread regressively to the preceding noun, strengthening the impression that the two signs form one prosodic unit. For example, in sentence (17), the signer raises her eyebrows for the sign glasses, but this facial expression starts on the preceding sign man. The result is that both signs are characterized by the same facial expression. The glosses of these 10 instances with reduced secondary predicates are provided in (9)–(18), where (12) contains two embedded predicates.

(9) woman, [man sit_170_] eye^∧^look-at_400_++. face look++ (1.1.04)

    ‘A woman looks at a sitting man. She looks at his face.’

(10) [man sit_190_] stand-up_320_. stand-up. (1.1.12)

    ‘A sitting man stands up. He stands up.’

(11) man, [one^∧^woman sit_70_], picture square, look show_260_. look (1.1.14)

    ‘A man shows a picture to a sitting woman. She looks.’

(12) [one^∧^girl small], [sit_280_ nervous_250_++ tear_350_++ paper].tear_270_ (1.1.15a)

      ‘A seated nervous girl is tearing some paper’, ‘A girl, seated and nervous, is tearing paper.’

(13) [woman house_110_ there_90_] run_590_++ go-path (1.1.18)

      ‘A woman in the house is running.’

(14) [one^∧^girl small sit_220_], cry_640_++ one (1.1.30)

      ‘A sitting girl is crying.’

(15) woman1, woman2 woman1, [sit_220_ legs-crossed_350_ roll_600_++] (1.5.07)^9^

      ‘A woman, sitting with her legs crossed, is rolling a ball.’

(16) mother. ix mother woman sit_420_. girl brush-other_450_++ brush_300_++, [mother sit_210_] (1.5.22)

      ‘A mother is sitting. A girl is brushing the sitting mother's hair with a brush.’

(17) man book cupboard [man glasses_270_], book put-in_340_ open-cupboard into (1.5.25)

      ‘A man with glasses is putting a book in a cupboard.’

(18) [ix man glasses_160_], book put-in_420_. arrange. put-in (1.6.25b)

      ‘A man with glasses is putting a book in. He's arranging things. He puts it in.’

We are now in a position to extend the analysis behind annotating (7) as (9) to examples (10)–(18). In all of these sentences, there are two signs that have a predicative function. But one of them is reduced in form and forms a constituent, either with a preceding nominal sign or, in the case of (12) and (15), with the following sign^10^. The phonological reduction is very clear. In the 10 cases, there was a marked difference between the duration of the two predicates: means of 214.8 ms for the short predicates, and 492.2 ms for the long predicates. Welch's two-tailed *t*-test suggests that this difference is robust, *t*_(14.89)_ = 5.45, *p* < 0.001. The phonological reduction, together with the prosodic cues indicating that the shorter predicate forms a constituent with the preceding nominal, are taken as evidence that the two predicative signs are of different grammatical status: the unreduced predicate is the main predicate in the clause, whereas the reduced predicate is a modifier of the preceding noun, an embedded predicate.

It is important to stress that not all responses containing two signs with a predicative function were analyzed as containing an embedded predicate. Compare clause (13) above (repeated here as 19) with (20);

(19) [woman house_110_ there] run_590_++ go-path (1.1.18)

      ‘A woman in the house is running.’

(20) woman, house_320_, walk-path, one (1.1.19)

      ‘A woman is walking in a house.’ or ‘A woman, in a house, is walking’

The two responses are almost identical in terms of the signs used. Yet their prosodic structure is different, signaling different constituent structure and consequently a difference in the function of house in the two clauses. In (19), house is very short (110 ms), it is signed with a single movement (the two hands touch each other once), and there is no hold or pause between woman and house. In (20), on the other hand, house is almost three times longer, 320 ms. long. It has a double movement (the hands touch each other twice), and there is a hold on woman, indicating a break between woman and house. This break prevents us from interpreting house as forming a constituent with woman; it forms its own prosodic constituent in the clause. Therefore, it is impossible to tell whether house modifies woman or walk-path, and both “the woman is walking in the house” and “the woman, in the house, is walking” are possible translations of this clause. Since there is no clear positive prosodic evidence for analyzing house as modifying woman, we did not regard this response as an instance of embedding.

As these examples show, not all of the embedded predicates, that is, signs in a modifier position, are signs denoting actions or events. The signs that we found in this position are as follows: seven occurrences of stative predicates (sit), one psych predicate (nervous), one locative noun (house) and two instances of an attributive noun (glasses). They function as modifiers of the preceding noun, and were interpreted according to the nature of the modifier: “a sitting/seated man,” “a nervous girl,” “a woman in a house,” “a man with glasses/a bespectacled man.”

How should these predicates be analyzed? If we draw on the translations, it is tempting to analyze them as participles or reduced relative clauses: “a girl (who is) sitting,” “a woman (who is) in the house,” “a man (with/who has) glasses.” Yet such a step is dangerous, since we are imposing the grammatical structure of one language (English) on another (KQSL). In English, participles have distinct morphological forms, and relative clauses are usually marked syntactically by a relative pronoun. In KQSL this is not the case. We have not found as yet any evidence for morphological markings that distinguish nouns from verbs, and no morphological evidence for the existence of participles. Furthermore (as is common in sign languages generally), we have not found any relativizers or other function words that mark subordination. It might be argued that the lack of evidence for such structures is due not to the simplicity in structure of the language but rather to the preliminary stage of investigation. However, studies of other village sign languages (e.g., ABSL, Aronoff et al., 2008; Padden et al., 2010, and other sign languages reported in Zeshan and de Vos, 2012) indicate that KQSL is not the exception; clear cases of syntactic manifestations of subordination have not been reported in other village sign languages. We are therefore reluctant to regard these reduced predicates as morphologically marked participles or bona fide subordinate clauses^11^.

However, the prosodic cues clearly show that we have clausal constructions with two predicate signs, one of which, reduced in form, occurs in a modifier position, and is perceived as secondary, that is, as modifying a referent rather than as the main predicate. We therefore suggest that these are cases of *embedding* of a predicate within a clause, in the very basic sense of the term. In the next section we describe a possible path of emergence for this construction, and examine its consequences for our understanding of the development of embedding in language.

## Discussion: embedding by hand and by mouth

As mentioned earlier, subordination and embedding are prevalent in the languages of the world. From a functional point of view, subordinate and main clauses are construed as cognitively asymmetrical (Langacker, 1987; Cristofaro, 2003). Functional asymmetry is usually reflected in the asymmetry of the form. The dependent status of a subordinate clause is often manifested in the deranking of its predicate through the use of a predicate form that is not used in independent clauses. The predicate deranking can be realized in different ways, for example, by the use of nominal markers on dependent predicates (nominalization), or through the lack of formal distinctions characteristic of independent predicates (Stassen, 1985; Croft, 1991; Cristofaro, 2003).

Diachronic studies show that subordinate structures often originate from simpler structures. Deutscher (2000), for example, traces the emergence of subordinate clauses in Akkadian, documenting the transition from parataxis of two adjacent clauses to full embedding of one in the other. Yet the question of how this process originates has proven difficult to answer without data on novel embedding structures in a given language.

### The diachronic development of subordination

In their study of grammaticalization, Heine and Kuteva (2007) and Heine (2009) suggest that there are two main paths in which subordinate clauses arise^12^. The first is through a process of expansion, by reinterpreting a nominal as clausal. The second is by integration of two independent clauses into one, where one of the main clauses becomes subordinate to another. Heine (2009) notices that the first strategy usually gives rise to complement embedded clauses, while relative clauses and adverbial clauses usually arise via the second strategy, integration. Regarding the expansion process, Heine suggests that the first stage toward subordination is the appearance of a non-finite verb form (nominalization, infinitive or participle) in a nominal position in the clause. In subsequent stages, the phrases headed by a non-finite form of the verb acquire more and more verbal properties, eventually becoming clausal.

Deutscher (2009) points out that the scenario described by Heine and Kuteva (2007) and Heine (2009), though plausible, misses a crucial point. According to Deutscher, the real syntactic-cognitive feat of subordination is nominalization, the ability to derive a noun from a verb. The expansion of a nominalized verb into a clause is a secondary development, which builds on a structure that already contains subordination, at least from a cognitive point of view. As Deutscher puts it, “The ability of a language to derive a noun from a verb, that is, to reify a verbal predicate and to present it as a nominal argument or modifier, is at the core of subordination.” (p. 199). While Heine takes the transition from “Stage 0,” which contains only nominal constituents, to “Stage 1,” which contains a nominalized verb in a constituent position, as the first step of subordination, Deutscher argues that the transition from Stage 0 to Stage 1 is what needs to be explained, since it cannot be taken for granted. For him it is this step, the appearance of a nominalized verb in a non-predicative position, that needs to be explained. Yet most accounts of the development of subordination have neglected to do so.

Deutscher then points out that in the grammaticalization literature, it is very hard to find works on the development of markers that signal V > N change. He attributes the difficulty of finding such grammaticalization paths to the absence of source constructions with a nominal head that takes a verb as its complement. Such constructions necessarily involve nominalization, and therefore already contain an instance of subordination. He speculates that backformation might be one route to nominalization. He considers the French suffix -*age* which was originally attached to nouns: *mari* “husband” + *age* = “the state of being a husband.” The denominal noun *mariage* was then reanalyzed as being derived from the verb *marier* “to marry,” rendering -*age* a nominalizing suffix that can attach to verbs.

A different approach to the development of embedded structures is offered by Mithun (2009), examining data from Mohawk. Mithun suggests that in Mohawk integration of two clauses can be done only by prosodic means, with no syntactic indication of subordination. Two clauses, one a semantic complement of the predicate of the other, or two clauses that share an argument, may occur under one intonational contour, as in example (21) below (Mithun, 2009, p. 60):

(21) [*Tóka' ki'     nèː'ne   kiː          iakoia'takarénie's*]

       Maybe just      it.is    this              bus

       [*t-hoti-ia'té-nha'        wáhi'*],

       carried.them.here             tag.question

           [*kiː    ratiksa'okònː'a]         thonéːnon       kènː'en*]

            this children              they.have.come       here

         ‘Maybe the bus brought the children that came here’.

Mithun argues that the clauses in (21) are pronounced under a single prosodic contour. Within this overarching contour the pitch moves from High at the beginning of the first clause (*Tóka'*) to a full terminal fall at the end of the last clause (*kènː'en*), with only partial pitch resets at the beginning of the internal prosodic phrases. She suggests that in this example one overall prosodic contour made up of small constituent sub-contours can be characterized as an instance of embedding. The author notices that the prosodic integration of the two clauses “reflects a kind of cognitive organization similar to that reflected by syntactic integration” (p. 61). That is, integration or embedding need not be reflected in the morpho-syntax; the cognitive feat of embedding is the ability to integrate two clauses in one construction. But this can be achieved by prosodic means alone in Mohawk. She further suggests that “The fact that we find prosodic structure without substantive syntactic structure suggests that prosodic structuring might, at least in some cases, precede syntactic structuring” (p. 61). Similar cases of subordination structures marked only by intonation contours have been reported of other languages as well, e.g., Bambara (Bird, 1968, cited in Givón, 2012), and several languages in the Niger-Congo area (Givón, 2012).

These two approaches taken together suggest that embedding is first and foremost a cognitive operation, the integration of one proposition within another. This can be done by morpho-syntactic means, such as the development of nominal forms of verbs, or by prosodic means, by uttering two clauses under the same prosodic contour. KQSL differs from the languages investigated by Deutscher and Mithun in that it has neither morpho-syntactic marking of clause integration nor of parts of speech^13^.

Studying the data from KQSL, we cannot claim that the secondary predicates we identified are nominal forms of verbs, since we have not discovered yet clear formal indication of parts-of-speech categorization in the language; all we can say is that they occur in a modifier position, function as modifiers, and have a form which can be regarded as less independent than the main predicates from phonological and prosodic points of view. However, we do think that KQSL offers a unique opportunity to look at the very first stage of embedding, the steps leading from Stage 0 to 1, that is, the possible initial stages of creating a subordinate predicate. Furthermore, since the only clues for the embedded status of these predicates is prosodic, KQSL bears witness to the role that prosody plays in the emergence of embedding.

A recent study on homesign (Hunsicker and Goldin-Meadow, 2012) may provide additional evidence as to how embedded constituents might arise in a communication system. The study describes the nominal constituents in the gestures of a young homesigner they call David. While David has iconic gestures (“nouns”) and pointing gestures (“determiners”), he has also been developing more complex nominal constituents comprising of both a noun and a determiner. For example, the string in (22) could be parsed and interpreted in two ways: “that is a bird and it pedals,” or the monoclausal “that bird pedals.”

(22) point-at-bird BIRD PEDAL

What Hunsicker and Goldin-Meadow show is that the monoclausal interpretation is more plausible, implicating the existence of embedded structure: [[point-at-bird BIRD] PEDAL]. Here is how the argument goes. First, they obtained the distribution of sentence lengths in David's production throughout the corpus without the sentences under examination, as measured in units (number of gestures). They then calculated two distributions of length: one with these sentences as “flat” structures, where each gesture corresponds to exactly one unit. In this case [[point-at-bird] [BIRD] [PEDAL]] would have three units. The other distribution was calculated with the hypothesized hierarchical structure, where [[point-at-bird BIRD] PEDAL] has two units. They found that the distribution with embedded structures provided a better fit to the data, leading them to conclude that the structures they investigate are to be seen as complex nominal constituents.

This case is remarkable since—as the authors discuss at length—David did not receive any structured hierarchical input from his caregivers. That he nevertheless developed embedded structure is in accordance with our findings as well; he managed to “squeeze” extra information into what was originally a basic nominal. One question that arises is how this was accomplished. We have provided evidence here that prosody is the resource used by KQSL signers to create embedded structure. Prosody is clearly co-opted in David's case as well: “Motoric criteria were also used to determine the end of a string of gestures and thus sentence boundaries. Two gestures were considered separate sentences if the child paused or relaxed his hands between the gestures. Gestures that were not separated by pause or relaxation of the hands were considered part of the same sentence.” (Hunsicker and Goldin-Meadow, 2012, p. 736). The authors do not provide a prosodic analysis, and it is wise to be cautious about attaching labels such as “prosody” to generalizations about rate of signing in the homesign system of a young child. However, research on sign languages leads us to expect timing breaks in particular places. For example, consider example (23), adapted from (6b) in Hunsicker and Goldin-Meadow (2012, p. 743). If David's production is sensitive to factors such as signing rate and cognitive complexity of constituents, we would predict a short prosodic break between LONG and *point-downstairs*.

(23) [[point-at-self point-at-paddle LONG] point-downstairs] ‘my long paddle is there’

In sum, it might be possible that what regulates the length of sentences and constituents in David's system reflects the emergence of a prosodic system, which in turn enables hierarchical structures that may lead to embedding. Naturally, more work is required in order to substantiate this claim, in village sign languages as well as in homesign.

### KQSL: a route toward embedding

The structure in question—a clause containing a secondary predicate—appears to be a new phenomenon in KQSL, not just because of its limited frequency but also in light of the fact that 9 of the 10 instances documented were produced by the two younger signers in our pool, those aged 42 and 44. Labov (1972) famously discussed the notion of “apparent time”: in the study of language variation and change, when two age groups vary on one sociolinguistic variable, it is likely that the older group represents the previous stage in the development of the language, and the younger group, the later stage. It may well be the case that embedding is an emerging phenomenon in KQSL, owing to its relative prevalence among younger signers as compared to older ones. Under the assumption that the phenomenon described here is indeed a new development in the language, we speculate on where it might have come from and hypothesize about its possible implications for the future of KQSL.

We have argued above that the secondary predicates appear in a position designated for a nominal modifier, the modifier of a noun that forms a syntactic constituent with it. The recurrence of postnominal modifiers in this position creates a construction with a specific form and function, that is, a *slot* for postnominal modifiers. A crucial development in the language, then, is the emergence of the modifier slot. Though the presence of a modifier position may seem self-evident, findings from KQSL teach us that it is a significant development. In the data that we have so far, consisting of 213 responses for the video clips, and three conversations between dyads of signers (total of 25 min), there are very few clear instances of nominal modifiers. Though KQSL has words denoting properties, e.g., good, bad, fast, new, they seem to function mostly as main predicates. The few clear examples of nominal modifiers are: picture square (“a square picture”), girl four (“four girls”), house small (“small houses”) and the forms female small (“a girl”) and mother big (“a woman/ an adult woman”). Of these examples, only the last two are common in our data (about 70 occurrences), and we hypothesize that they are lexicalized collocations or compounds. These signs, then, clearly have a referential (and not a predicative) function, and they may have paved the way to the creation of a modifier slot^14^.

Once a modifier slot is available, it can be used by various types of words. In our data we find locatives (woman house there “a woman [who is] in a house there”) and nouns denoting attributes (man glasses “the man with the glasses”). We also find signs denoting stative (stage-level) predicates such as nervous and sit.

We might speculate that a possible developmental path for the existing forms may have been moving from simple adjectives, such as “small” and “big,” to locatives like “house” and then statives like “sitting.” The next logical step would be the introduction of non-stative verbs such as perhaps “look” or “talk” in that slot. Such verbs can introduce complements, possibly leading to the appearance of clausal constituents in the modifier slot. However, this will require the development of more grammatical machinery. What signals predicates as secondary in our data is prosody: these signs tend to form one prosodic unit with the noun they modify, and are reduced in form. But if the embedded predicates take their own complements, they will probably form another prosodic unit, maybe separate from the head noun. In such a case, the language will need to develop a grammatical mechanism to mark this predicate and its complements as secondary. Some sign languages do this by means of facial expressions; in ISL, for example, relative clauses are often marked with a squint (Dachkovsky, 2008; Meir and Sandler, 2008; Dachkovsky and Sandler, 2009; Dachkovsky et al., 2013). Yet recruiting facial expressions for grammatical purposes takes time to develop; it is not there in early stages of a language (Sandler et al., 2011; Sandler, 2013). KQSL shows us that an initial step toward developing a relative clause is the creation of a modifier slot that can host different types of signs. The next step, the accommodation of clausal material such as arguments and adverbials in this slot, has not yet been attested.

## Conclusion

This study has showcased a certain phenomenon in KQSL in order to shed light on the question of embedding in the evolution of natural language. Some younger adult signers of KQSL seem to use the postnominal modifier position as a slot open to several types of modifiers that can then act as secondary predicates. Locatives, concrete objects, psych predicates and stative predicates have been recruited as nominal modifiers. We have speculated that these developments might continue, with the embedded structures gradually allowing more elements and eventually leading to full-fledged relative clauses. Future work on KQSL will be needed to determine whether the hypothesis regarding the expansion of postnominal slots to host larger structures turns out to be correct.

Embedding, or, more specifically, recursion, has been at the core of recent debate on the nature of the human language faculty (see the debate in e.g., Hauser et al., 2002; Pinker and Jackendoff, 2005; Jackendoff, 2011; Watumull et al., 2014). While our work does not take a stand on this issue, it does provide evidence for an early stage of embedded structures in a natural language. Our data show that semantic embedding, that is, the embedding of a predicate within another, might be there from very early on in the development of a language, although the grammatical machinery to accommodate and mark embedding takes time to develop. Moreover, the earliest form of such grammatical machinery may not be clearly syntactic or morphological, but rather prosodic. The prosodic structure marks constituent boundaries of different degrees, signaling, inter alia, constituents within constituents, as in our case^15^. KQSL suggests that the initial stages of embedding may be quite modest: the creation of a slot for modifiers. Yet this modest step is crucial to get the wagon moving. Deutscher (2009, p. 212) argues that “Any attempt to explain the genesis of subordination can thus only begin to make sense if it explains the origins of nominalization, and if it shows how the ability to repackage a verb as a noun arises in contexts where it had not existed before.” Our work shows how such repackaging might have started to develop. In a very young language such as KQSL, which hasn't developed morphological markings for nominalization or syntactic mechanisms for subordination, such repackaging is done by means of prosody: predicates are “squeezed” into a modifier position, becoming embedded. The various predicates found in this structure further suggest that even such a humble step is composed of sub-steps, where more prototypical modifiers, such as *big/small*, may have paved the road to other, less typical, and even verbal predicates. KQSL enables us to zoom in on these initial stages, which are very hard to come upon when investigating the origins of subordination in spoken languages. The relative newness of sign languages compared to spoken languages makes them indispensable for our understanding of how linguistic structures arise and develop.

### Conflict of interest statement

The authors declare that the research was conducted in the absence of any commercial or financial relationships that could be construed as a potential conflict of interest.

## Footnotes

^1^The ELAN tool was developed at the Language Archive, Max Planck Institute for Psycholinguistics, Nijmegen (http://tla.mpi.nl/tools/tla-tools/elan/). See Crasborn and Sloetjes (2008) for a discussion of the use of ELAN to code sign language data.

^2^For differences prosodic uses of head movement in ISL and ASL, see Dachkovsky et al. (2013).

^3^Unlike the other criteria that we used, which are all supported in detail by earlier research, facial expression spread from a specific word has not been employed in earlier work and requires further confirmation. However, the criterion is lent credence by its similarity to another previously supported phenomenon: the mouthing of spoken words in ISL, which spreads from the specific sign it accompanies to neighboring signs within small prosodic units (Sandler, 1999).

^4^The emergence of a prevalent word order does not mean that all clauses in KQSL contain a straightforward arrangement of subject, object and verb. See examples (16) and (17) below for utterances in which some arguments or predicates are repeated.

^5^Following convention, we use small caps to denote glosses of individual signs. The caret ^∧^ is used to denote compounding. Multiple plus signs ++ denote repetition. A hyphen indicates that two words in the gloss correspond to one sign. For example, the two words look-at correspond to one sign in KQSL. Full stops indicate clausal intonation break, and commas indicate minor intonational breaks, that is, intonational breaks within a clause. Square brackets [ ] delimit the prosodic constituents whose position and length are relevant for our analysis, as described in the text. Some signs have different versions (synonyms), and these are glossed as e.g. woman1 woman2. ix denotes an index (pointing in space). Each utterance is followed by its identifier in our database. Where relevant, sign duration in milliseconds is given as a subscript.

^6^The sign LOOK-AT is signed with a pointing sign towards the eye and then a V hand moving outwards. They form one lexical unit, “look-at.”

^7^In the videotape of the sentence illustrated in Figure 7, the dominant hand is beginning to transition from the handshape for MAN (fist) to the shape for SIT (flat, open hand), while still at the location for MAN, at the same time that the nondominant hand moves toward the position for SIT. The coarticulatory movement was so fast that only a blurred transitional shape was visible, and we elected to represent the handshape of the sign MAN in Figure 7, rather than the blurred transition, for clarity.

^8^Example (15) below is slightly different, as explained in fn. 9.

^9^In this example, the embedded predicate does not form one constituent with the preceding head noun. This might be a result of the fact that the head noun is a phrase consisting of three lexical nouns (woman1, woman2, woman 1), and the signer is a bit hesitant as to which sign of “woman” to use. The embedded predicate also consists of two signs (sit legs-crossed). Since both constituents consist of more than one sign, they each form a separate prosodic unit. However, the sign sit is still very short, which we take to indicate that it is not the main predicate in the clause.

^10^In these two examples (12 and 15), the reduced predicate itself is being modified, by nervous and legs-crossed respectively. In addition, the constituent referring to the subject in these examples contains more than one sign. The prosodic break after the subject constituent might be due to the relative “heaviness” of this constituent in these examples. However, we regard these clauses as instances of embedded predicates rather than two coordinate clauses (e.g. “a woman sits cross-legged and rolls a ball”) because of the clear difference in duration and size between the predicates in the clauses.

^11^The literature on relative clauses in established sign languages suggests that in some languages relative clauses contain function words that occur at the boundaries of relative clauses. In some sign languages these signs are optional (e.g. the sign that in ASL, Liddell, 1978, and a sign referred to as “relative pronoun” in Turkish Sign Language, Kubus and Rathmann, 2011). In other sign languages the use of a function word as a marker of relative clauses is reported as “systematic,” though the specific details and analyses vary across languages. See Pfau and Steinbach (2005) for German Sign Language, Branchini and Donati (2009) for Italian Sign Language, and Tang et. al. (2010) for Hong Kong Sign Language.

^12^The purpose of Heine (2009) is to show how devices that first served to structure independent sentences come to assume functions of subordination, and not necessarily to demonstrate the origin of subordination.

^13^The KQSL situation is somewhat reminiscent of initial stages of language development in children, when the parts-of speech-distinction is not fully established yet. Givón (2009), in a longitudinal study of the development of relative clauses, shows that children go through a stage in which they use various types of non-clausal post nominal modifiers, as in the following example: (i) MOT: I like rabbits, don't you? NIN: Yup. I like them. Like this one the red. MOT: You like red rabbits? NIN: Yup. The adjective *red*, modifying the noun *rabbit*, fulfills a restrictive function and occupies the post-nominal slot that at a later stage is taken up by relative clauses. In Givon's opinion, those constructions may be considered early precursors of standard relative-clause forms. The KQSL examples are similar in that post nominal modifiers take the function of relative clauses without developing specific syntactic structures for marking relativization as yet. As a reviewer points out, English constructions such as *the man I saw* can also be considered cases of prosodically-marked embedding.

^14^Deutscher (2005, appendix 3) speculates on the possible steps towards the emergence of a modifier slot in the development of a language. He suggests that deictic words such as demonstratives may have been the first words to have a modifier function, and by that they paved the way to create a modifier slot in the sentence structure. Our suggestion is compatible with Deutscher's hypothetical scenario, but we think that KQSL actually provides concrete data towards regarding the development of a modifier slot.

^15^As a reviewer points out, this classifies KQSL as a language with a “prosodic simple phrase grammar” in the terminology of Jackendoff and Wittenberg (in press).
