# Supplementary material for: The emergence of embedded structure: insights from Kafr Qasem Sign Language
Source: Front Psychol. 2014 Jun 3;5:525. doi: 10.3389/fpsyg.2014.00525 (PMC4042080; doi:10.3389/fpsyg.2014.00525)
Supplement: Supplementary file 1 [file DataSheet1.DOCX]

1. **Appendix A**

Concepts use in the vocabulary comparison.

| a lot | a little bit | afraid | angry |
| --- | --- | --- | --- |
| apple | ask | baby | bad |
| bed | Bedouins | bird | black |
| blue | bread | bus | butter |
| buy | car | car technician | cat |
| chair | cheap | clean | clean |
| coat | coffee | cold | complain |
| cow | cry | cucumber | dance |
| day | deaf | die | difficult |
| dirty | doctor | dog | door |
| drink | eat | egg | Egypt |
| end | expensive | family | far |
| fast | fat | father | female |
| fire | fish | forget | Friday |
| friend | Gaza | give | give birth |
| goat | gold | good | grandparent |
| happy | hat | healthy | hearing |
| horse | hospital | hot | house |
| how much? | hungry | important | Jerusalem |
| Jordan | kitchen | knife | laugh |
| learn | Lebanon | lemon | look for |
| love | male | man | maybe |
| meat | milk | Monday | money |
| month | moon | morning | mother |
| my | near | new | night |
| no | nurse | oil | old |
| old | olive | onion | orange |
| Palestinian authority | pants | phone | pig |
| poor | pray | pretty | rain |
| red | remember | rest | rich |
| river | run | sad | salt |
| same | Saturday | school | sea |
| seamstress | see | sell | sheep |
| Sheick | shoes | short | shout |
| sibling | sick | sit | slow |
| smart | sorry | stand | stop |
| street | strong | stupid | sugar |
| Sulha | sun | Sunday | Syria |
| table | tea | teacher | teaspoon |
| ten | think | thirsty | thousand |
| Thursday | tired | today | tomato |
| train | tree | truck | Tuesday |
| TV | twenty | ugly | understand |
| wait | wash(ones'self) | water | wedding |
| Wednesday | week | what? | when? |
| white | window | woman | work |
| worker | year | yellow | yes |
| yesterday |  |  |  |

1. **Appendix B**

Elicitation sentences

| 1 | A woman puts box on table |
| --- | --- |
| 2 | A woman gives a shirt to man |
| 3 | A girl drags a shopping cart |
| 4 | A woman looks at man |
| 5 | A bottle falls |
| 6 | A girl falls |
| 7 | A woman rolls a ball |
| 8 | A woman takes a pair of scissors from a girl |
| 9 | A man taps on watermelon |
| 10 | A girl pulls a man by the arm |
| 11 | Water spills |
| 12 | A man stands up |
| 13 | A girl runs in circles |
| 14 | A man shows a picture to a woman |
| 15 | A girl tears a sheet of paper |
| 16 | A woman pushes a girl |
| 17 | A plastic bag floats in the air |
| 18 | A woman runs |
| 19 | A woman walks |
| 20 | A man throws a ball to a girl |
| 21 | A man washes dishes |
| 22 | A girl brushes a woman's hair |
| 23 | A ball bounces |
| 24 | A man sleeps |
| 25 | A man puts a book on a shelf |
| 26 | A girl feeds a woman |
| 27 | A woman writes on a piece of paper |
| 28 | A man taps a girl on the shoulder |
| 29 | A ball rolls |
| 30 | A girl cries |
